# Higher Inpatient Morbidity and Mortality in Biliary Pancreatitis Compared to Hypertriglyceridemia-Induced Pancreatitis: A Nationwide Retrospective Study

**DOI:** 10.7759/cureus.10351

**Published:** 2020-09-10

**Authors:** Hafeez Shaka, Jennifer C Asotibe, Ikechukwu Achebe, Garima Pudasaini

**Affiliations:** 1 Internal Medicine, John H. Stroger, Jr. Hospital of Cook County, Chicago, USA

**Keywords:** hypertriglyceridemia, acute pancreatitis, biliary pancreatitis, mortality, gallstone pancreatitis

## Abstract

Introduction

Hypertriglyceridemia (HTG)-induced pancreatitis is the third most common cause of acute pancreatitis after gallstone disease and alcohol. We analyzed data from the National (Nationwide) Inpatient Sample (NIS) with the aim of evaluating the outcomes of patients with HTG-induced pancreatitis when compared to those with biliary-induced pancreatitis.

Methods

The NIS database was sourced for data involving adult hospitalizations for HTG-induced pancreatitis in the United States between January 1, 2016 and December 31, 2017. The main outcome was mortality in patients with biliary pancreatitis vs HTG pancreatitis. Secondary outcomes were the incidence of sepsis, septic shock, non-ST-elevation myocardial infarction (NSTEMI), blood transfusion requirements, acute kidney failure, acute respiratory distress syndrome (ARDS), and length of hospital stay.

Results

A total of 575,230 patients were admitted with a diagnosis of acute pancreatitis, 18.2% of which were classified as having HTG pancreatitis. The in-hospital mortality for pancreatitis was 0.59%. Patients with HTG pancreatitis had lower odds of in-hospital mortality (adjusted odds ratio [aOR]: 0.74, 95% CI 0.582-0.934, p=0.012) compared to those with biliary pancreatitis. Patients with HTG pancreatitis had less odds of developing comorbid sepsis (aOR: 0.52, 95% CI 0.441-0.612, p<0.001), septic shock (aOR: 0.64, 95% CI 0.482-0.851, p<0.001), and NSTEMI (aOR: 0.70, 95% CI 0.535-0.926, p<0.001) and had less odds of requiring transfusion of blood products (aOR: 0.57, 95% CI 0.478-0.678, p<0.001) when compared to those with biliary pancreatitis. Patients with HTG pancreatitis also had a lower average length of hospital stay and lower total hospital charges compared to those with biliary pancreatitis. There was no statistical difference, however, in acute kidney failure and ARDS between the two groups.

Conclusion

Patients with HTG-induced pancreatitis possibly have better inpatient outcomes including mortality when compared to those with biliary-induced pancreatitis.

## Introduction

Hypertriglyceridemia (HTG)-induced pancreatitis is the most common cause of acute pancreatitis after gallstone disease and alcohol abuse [1,2]. The incidence of HTG pancreatitis has been variously reported; however, most recent literature suggests the incidence to range from 7% to as high as 38% of patients presenting with acute pancreatitis [2-4]. This proportion increases in pregnancy, with HTG causing nearly 50% of acute pancreatitis cases in pregnancy [5,6]. While some patients inherit genes that predispose to HTG, others acquire the disease as a consequence of their obesity, diabetes, pregnancy, hypothyroidism, and medications [7-12].

Disease pathophysiology of HTG-induced pancreatitis is still being understood; however, accumulation of lipotoxic triglyceride metabolites, specifically free fatty acids, is thought to injure pancreatic tissue and cause subsequent inflammation. While triglyceride levels of above 1,000 mg/dl are thought to increase the risk of acute pancreatitis, the true level of HTG at which pancreatitis occurs is unknown [5,13].

Gallstone disease is responsible for up to 70% of acute pancreatitis cases and surpasses HTG as the leading cause of acute pancreatitis [3-7]. Gallstone obstruction of the ampulla causes restricted outflow of pancreatic secretions and occasionally bile reflux into the pancreatic duct leading to irritation and inflammation of the pancreas. Clinically, the presentation of gallstone pancreatitis is indistinguishable from HTG-induced pancreatitis. Both patients present with severe, epigastric abdominal pain, and associated nausea and vomiting. Demographically, gallstone pancreatitis has been found to be more common in females, while HTG-induced pancreatitis presents earlier than gallstone pancreatitis [14-18].

To date, studies investigating morbidity and mortality outcomes between HTG and gallstone pancreatitis are infrequent and inconclusive. While some studies report increased morbidity, mortality, and pancreatitis severity in patients with HTG pancreatitis compared to gallstone-induced disease, other studies have found no difference between the two groups [1,13,19-22]. To the best of our knowledge, this study represents the first large-scale nationwide study evaluating outcomes of HTG-induced pancreatitis compared to biliary-induced pancreatitis.

## Materials and methods

Study design

The present study is a retrospective cohort study that included adult admissions for HTG pancreatitis in the United States that occurred between January 2016 and December 2017. Data from the calendar years 2016 to 2017 were extracted from the NIS database and used in this study. The National (Nationwide) Inpatient Sample (NIS) provides data from inpatient hospital admissions that are acquired through billing data submitted to statewide organizations. Currently, the NIS covers over 97% of the population across 47 U.S. states and the District of Columbia, and has been shown to provide reliable inpatient estimates of comorbidity and disease prevalence [23,24]. International Classification of Diseases (Tenth Revision) (ICD-10), and Clinical Modification Procedure Coding System (CM/PCS) were used to conduct this study. The NIS utilizes two diagnosis categories, principal and secondary. The principal diagnosis represents the pathology that caused admission, while secondary diagnosis refers to all other assigned diagnoses [24].

Study population

NIS data were gathered from years 2016 through 2017 for patients aged 18 years and above who had a principal discharge diagnosis of acute pancreatitis (K85). Since there is no specific ICD-10 code for HTG pancreatitis, we formed this group by combining patients with a principal diagnosis of acute pancreatitis, not due to gallstones, alcohol, or drugs, and matched them with a secondary diagnosis of HTG, including pure hyperglyceridemia (E78.1), mixed hyperlipidemia (E78.2), hyperchylomicronemia (E78.3), other hyperlipidemia (E78.4), hyperlipidemia unspecified (E78.5), and lipoprotein deficiency (E78.6). A comparison group of patients with biliary acute pancreatitis (K85.1) along with patients with either idiopathic or unknown etiology of acute pancreatitis with concomitant cholelithiasis or cholecystitis (K80, K81) was generated to compare outcomes. Patients were excluded if they were younger than 18 years of age, had acute pancreatitis as a secondary diagnosis, or pancreatitis caused by other etiologies [24].

Study outcomes

The primary outcome was to compare inpatient mortality among patients admitted for HTG vs biliary acute pancreatitis. Secondary outcomes in this population include the development of sepsis, septic shock, non-ST segment elevation myocardial infarction (NSTEMI), need for transfusion of blood products, acute kidney failure, acute respiratory distress syndrome (ARDS), as well as mean length of hospitalization and mean total hospital charges.

Statistical analysis

Data were analyzed using the statistical software Stata® Version 16 (Stata Corp, College Station, TX, USA). All data analyses performed in this study utilized the Healthcare Cost and Utilization Project (HCUP) regulations NIS. Chi-squared testing was employed to compare characteristics between patients with HTG pancreatitis and other etiologies of acute pancreatitis [24]. Multivariate regression analysis was then used to remove the effect of possible confounders, and also to calculate outcomes (primary, and secondary). Univariate screening was utilized to confirm factors that had an effect on outcomes. Variables with a p-value of less than 0.2, were included in multivariate regression analysis. A p-value of less than 0.05 was our indication of statistical significance.

Ethical considerations

In an effort to protect patient information, the NIS has removed all possible patient and or hospital identifiers. With patient identifiers removed from data, this study did not require Institutional Review Board approval [24].

## Results

Patient characteristics

The combined NIS database for 2016 and 2017 contained over 71 million weighted hospital discharges; of which 575,230 have a principal discharge diagnosis of acute pancreatitis [24]. Of these patients, 18.2% were classified as having HTG pancreatitis, while the other subgroup comprises various other etiologies of acute pancreatitis.

Patients with HTG pancreatitis were significantly older (56.7 vs 50.8 years, p<0.001), and had a higher proportion of whites and Hispanics. These patients had more comorbidities, including diabetes (52.6% vs 20.6%, p<0.001), obesity (24.6% vs 15.2%, p<0.001), hypothyroidism (13.1% vs 7.9%, p<0.001), chronic ischemic heart disease (21.7% vs 9.1%, p<0.001), and hypertension (60.3% vs 43.9%, p<0.001) compared with patients with other etiologies of acute pancreatitis. Patients with HTG pancreatitis had a lower proportion of prior tobacco use. Patient and hospital characteristics are detailed in Table 1.

**Table 1:** Distribution of Patient and Hospital Characteristics for Acute Pancreatitis Admissions: Non-HTG vs HTG #: for 2017 CHF, congestive heart failure; CKD, chronic kidney disease; COPD, chronic obstructive pulmonary disease; CVA, cerebrovascular accident; HTG, hypertriglyceridemia; IHD:, ischemic heart disease

Variable (n=575,230)	Non-HTG pancreatitis, n=470,765 (81.8)%	HTG pancreatitis, n=104,465 (18.2)%	P-value
Patient characteristics			
Age, mean	50.8	56.7	<0.001
Women	47.2	45.5	<0.001
Racial distribution			<0.001
White	61.6	64.4	
Black	17.3	12.9	
Hispanic	12.0	13.3	
Others	9.1	9.4	
Insurance type			<0.001
Medicaid	29.5	43.6	
Medicare	27.1	17.4	
Private	33.3	33.3	
Uninsured	10.1	5.7	
Charlson Comorbidity Index score			<0.001
0	45.1	21.6	
1	29.0	33.5	
2	12.3	20.7	
≥3	13.6	24.2	
Median annual income in patient’s zip code, US$^#^			0.033
1-43,999	33.2	32.4	
44,000-55,999	26.5	27.4	
56,000-73,999	23.1	23.4	
≥74,000	17.2	16.8	
Comorbidities			
Diabetes	20.6	52.6	<0.001
Hypertension	43.9	60.3	<0.001
Smoking history	48.3	45.4	<0.001
CHF	5.0	8.1	<0.001
CKD	6.1	11.0	<0.001
Hypothyroidism	7.9	13.1	<0.001
Obesity	15.2	24.6	<0.001
Chronic IHD	9.1	21.7	<0.001
Prior CVA	0.7	1.2	<0.001
COPD	9.0	12.2	<0.001
Anemia	17.4	17.1	0.217
Hospital characteristics			
Hospital region			<0.001
Northeast	17.1	15.6	
Midwest	22.3	24.1	
South	40.5	42.6	
West	20.1	17.7	
Hospital bed size			0.003
Small	23.6	22.4	
Medium	30.7	30.8	
Large	45.7	46.8	
Urban location	88.1	86.8	<0.001
Teaching hospital	60.2	58.9	0.002

Primary outcome: in-hospital mortality

The in-hospital mortality for patients with acute pancreatitis was 0.59%. Patients with HTG pancreatitis had a lower odds of in-hospital mortality (adjusted odds ratio [aOR]: 0.74, 95% CI 0.582-0.934, p=0.012) compared to those with biliary pancreatitis when adjusted for comorbidities (age, sex, racial distribution, Charlson disease severity index, hospital location, hypertension, smoking history, chronic kidney disease, congestive heart failure, obesity, diabetes, chronic ischemic heart disease, chronic obstructive pulmonary disease, hypothyroidism, and liver disease) using multivariate logistic regression analysis.

Secondary outcomes

Patients with HTG pancreatitis had less odds of having comorbid sepsis (aOR: 0.52, 95% CI 0.441-0.612, p<0.001), septic shock (aOR: 0.64, 95% CI 0.482-0.851, p<0.001), NSTEMI (aOR: 0.70, 95% CI 0.535-0.926, p<0.001), and transfusion of blood products (aOR: 0.57, 95% CI 0.478-0.678, p<0.001) compared to those with biliary pancreatitis. Patients with HTG pancreatitis also had a lower mean length of stay and mean total hospital charges compared to those with biliary pancreatitis.

There was no statistical difference in comorbid acute kidney failure and ARDS between the two groups. Detailed outcomes are provided in Table 2.

**Table 2:** Clinical Outcomes of Acute HTG Pancreatitis Compared to Biliary Pancreatitis *, statistically significant; #, adjusted mean difference; HTG, hypertriglyceridemia; aOR, adjusted odds ratio; CI, confidence interval; NSTEMI, non-ST segment elevation myocardial infarction

Outcome	Biliary pancreatitis	HTG pancreatitis	aOR (95% CI)	P-value*
Primary outcome %				
In-hospital mortality	0.8	0.6	0.74 (0.582 to 0.934)	0.012*
Secondary outcomes %				
Length of stay, mean	5.1	4.1	0.9 (-1.0 to -0.8)^#^	<0.001*
Total hospital charges, mean US$	54,717	34,337	-19,331 (-20,597 to -18,065)^#^	<0.001*
Sepsis	2.3	1.1	0.52 (0.441 to 0.612)	<0.001*
Septic shock	0.7	0.4	0.64 (0.482 to 0.851)	<0.001*
NSTEMI	0.6	0.4	0.70 (0.535 to 0.926)	<0.001*
Blood transfusion requirement	2.0	1.1	0.57 (0.478 to 0.678)	<0.001*
Acute kidney failure	11.9	13.3	1.02 (0.957 to 1.088)	0.545
Acute respiratory distress syndrome	0.2	0.2	0.78 (0.500 to 1.229)	0.289

## Discussion

Acute pancreatitis is a recognized cause of increased morbidity and mortality in hospitalized patients and leads as a top cause of gastrointestinal related admissions. With continued increases in population obesity, poor dieting, and gallstone disease the national incidence of acute pancreatitis is expected to rise [4,5]. Our study is one of the few large-scale and nationwide cohort studies to evaluate outcomes of HTG-induced pancreatitis compared to biliary-induced pancreatitis.

Compared to other studies like Deng et al., who reported increased incidence of multiorgan failure, shock, renal failure, and overall mortality in patients admitted with HTG, our study found patients with HTG pancreatitis to have lower odds of in-hospital mortality compared to those with biliary pancreatitis (aOR: 0.74, p=0.012) [19,25]. Additionally, patients in the HTG pancreatitis cohort had lower adjusted odds of severity indices, which included sepsis, septic shock, NSTEMI, and need for transfusion requirements. This finding differs from current literature which has found either no statistically significant difference between HTG-induced pancreatitis and other causes of pancreatitis, including gallbladder disease, or higher disease severity in patients with HTG-induced pancreatitis [4,13,16,19,20,25].

Compared to studies that tested this data in specific populations, or using smaller sample sizes, our study conducted a large nationwide analysis of data which increases the power of the study and renders it less prone to selection bias. In agreement with our study finding, another large-scale population-based study investigating HTG-induced pancreatitis found lower mortality in patients with acute pancreatitis and HTG. These findings lend credibility to the possibility that the commonly accepted idea of HTG-induced pancreatitis being associated with increased disease severity when compared to other causes of pancreatitis is incorrect [26].

We postulate that the comparatively reduced morbidity and mortality seen in HTG pancreatitis compared to gallstone-induced pancreatitis is a consequence of the increased utilization of invasive management for patients with biliary pancreatitis. Due to the high recurrence rate of biliary pancreatitis that occurs when patients do not undergo definitive management, strategies to manage gallstone pancreatitis have shifted towards performing necessary interventions (endoscopic retrograde cholangiopancreatography and cholecystectomy) before patient discharge [27,28]. Although necessary, these procedures increase the risk of complications including sepsis, septic shock, NSTEMI, and increased blood transfusion requirements. Compared to HTG-induced pancreatitis which typically is managed conservatively, the need for invasive procedures in the management of gallstone pancreatitis theoretically should be associated with higher hospitalization charges and increased length of hospitalizations. This postulation was supported by our finding of shorter hospital stay and lower total hospital charges in patients with HTG-induced pancreatitis compared to gallstone-induced disease [2,6,17,29].

In contrast to a study carried out by Sekimoto et al., in which a younger overall age was reported in patients with HTG-induced pancreatitis, our cohort of patients with HTG-induced pancreatitis was older than the non-HTG cohort (56.7 vs 50.8 years, p<0.001) [14]. Our study also demonstrated that patients with HTG-induced pancreatitis had a higher proportion of comorbid diabetes, obesity, and hypothyroidism when compared to the non-HTG cohort.

Taking the results of this study into consideration, patients with gallstone pancreatitis possibly have more severe pancreatitis when compared to HTG-induced disease, and thus warrant close monitoring for the development of sepsis, shock, NSTEMI, and transfusion dependence, specifically when postinvasive intervention.

The limitations of this study are as follows. Despite being amongst the three leading causes of acute pancreatitis, there is no dedicated ICD-10 code for HTG-induced pancreatitis, making data query a challenge. This obstacle was overcome by excluding other known causes of acute pancreatitis from the study population and then extracting patients with diagnoses of HTG. Secondly, the limitations of the NIS database did not allow us to stratify levels of HTG and measure the possible effect it may have on outcomes. Given the fact that the level of HTG has not been shown to correlate with disease severity in current literature, our concerns were partially remedied. Lastly, the retrospective nature of this study establishes associations but does not imply causality.

Despite these limitations, our large sample size, scientific questioning, and analysis technique help contribute new information to a largely understudied topic of outcomes of HTG-induced pancreatitis when compared to gallstone pancreatitis. Through this study, we hope to increase clinician suspicion for pancreatitis-related complications and look to encourage more large-scale investigations.

## Conclusions

Large-scale studies that comparatively investigate morbidity and mortality outcomes in HTG and gallstone-induced pancreatitis are infrequent. While some studies have found no difference in outcomes, other smaller-scale studies suggest increased disease severity in HTG-induced pancreatitis. To the best of our knowledge, this study represents one of few large-scale, nationwide studies to evaluate outcomes of HTG-induced pancreatitis compared to biliary-induced pancreatitis. Results from our study suggest that patients with HTG pancreatitis possibly have better inpatient outcomes including mortality compared to those with biliary pancreatitis. Taking the results of this study into consideration, patients with gallstone pancreatitis warrant close monitoring for the development of sepsis, shock, NSTEMI, and transfusion dependence, especially after invasive procedures.
